# Coping Styles Predict Timing of Dementia Diagnosis: Evidence From DETERMIND

**DOI:** 10.1002/gps.70246

**Published:** 2026-07-31

**Authors:** Isaac Duncan‐Cross, Eleanor Miles, Ben Hicks

**Affiliations:** ^1^ University of Sussex Brighton UK; ^2^ University of Nottingham Nottingham UK

**Keywords:** alzheimer's disease, coping styles, dementia, DETERMIND, diagnostic timing, help seeking

## Abstract

**Objectives:**

Early diagnosis underpins global policy directives aimed at supporting people to live well with dementia. While people encounter numerous barriers when seeking early dementia diagnosis, previous research has primarily focussed on non‐modifiable, socio‐demographic determinants of diagnostic timing. This study examined whether individuals' coping style could provide further insight into diagnostic delay.

**Methods:**

Coping was assessed via the dispositional Brief COPE in 935 individuals recently diagnosed with dementia and 697 carers from the DETERMIND cohort. Through principal component analysis, we identified three distinct coping styles. Hierarchical regression assessed the association of participant demographics and coping factors with diagnostic timing, operationalised via symptom severity and time between first symptoms and diagnosis.

**Results:**

Coping predicted diagnostic timing in terms of both symptom severity and temporal delay. Increased symptom severity at diagnosis was predicted by individuals with dementia using more avoidant coping and less approach coping. Longer temporal delays were associated with individuals with dementia using less support seeking coping and carers using less avoidant coping.

**Conclusions:**

Our findings suggest diagnostic timing is related to the coping strategies employed by individuals with dementia and their carers, over and above demographic influences. These results identify coping as a modifiable factor that could help understand who is most at risk and inform interventions to promote early diagnosis, aligning with global policy directives.

## Key Points

- Diagnostic timing was significantly predicted by the coping styles of people with dementia and their carers, over and above traditionally researched demographic variables.
- For people with dementia, using more approach coping and less avoidant coping predicted reduced symptom severity at diagnosis while increased support seeking was associated with shorter temporal delays.
- For caregivers, shorter temporal delays (time between symptom onset and recognition) were associated with increased avoidant coping.
- Our results highlight coping styles as a modifiable psychological factor, meaningfully associated with diagnostic timing in people with dementia.

## 1 Background

Early diagnosis for individuals with dementia is a key healthcare goal [1]. An early diagnosis can allow individuals to live better for longer through accessing support services and symptom alleviating treatments, as well as providing time to plan for the future [2]. Furthermore, modelling studies suggest early diagnosis also provides societal economic benefits by reducing reliance on more expensive care or institutionalisation [3]. However, despite most individuals with dementia wishing to know their diagnosis at the earliest opportunity [4], rates of underdiagnosis remain high. Around 75% of people estimated to currently have dementia do not have a diagnosis [5], while those who do typically receive them several years after first noticing symptoms [6].

Diagnostic timing may be influenced by multiple factors, including individuals' symptom awareness, demographics, and practical or institutional barriers [7, 8, 9]. However, most of these factors cannot explain why many individuals choose to delay seeking support even after recognising their symptoms. One underexplored factor is the role of coping. The onset of dementia presents both practical and emotional challenges, prompting individuals to adopt coping strategies to manage their experiences [10]. These strategies, shaped by individuals' broader coping styles [11], vary widely and may play a key role in understanding and predicting diagnostic timing.

The relationship between coping and diagnostic timing has been highlighted in qualitative research. These studies have suggested that maladaptive coping strategies, such as avoidance, denial, and normalisation, may delay symptom acknowledgement and acceptance as well as engagement with diagnostic services [12, 13, 14]. Quantitative work, though sparse and mostly involving populations without dementia, indicates that coping may predict engagement with genetic risk testing [15, 16] and advanced care planning discussions [17]. Among caregivers, coping style also appears to influence the diversity and quantity of support they use [18] but not the service use of individuals with dementia [19]. Overall, this literature suggests coping may be an important factor in understanding why people with dementia may choose not to seek diagnosis, but no previous studies have statistically examined the role of coping in relation to diagnostic timing.

To address this evidence gap, the present study aimed to examine whether coping styles for people with dementia and their carers could predict diagnostic timing. If so, coping could represent a novel means for identifying those most at risk of delays and a valuable target for future interventions and policies aimed at promoting earlier diagnosis.

## 2 Materials and Methods

### 2.1 Recruitment

This study used data from participants in DETERMIND [20], a prospective cohort study examining inequalities in dementia care and outcomes. Recruitment began in August 2019 and ended in March 2023, with a year hiatus from March 2020 to March 2021 during the COVID‐19 pandemic. Individuals were recruited within 6 months of receiving a clinical diagnosis of any dementia, along with their primary carers where possible. Individuals were recruited from Memory Assessment Services within Southeast England, London and Northeast England.

### 2.2 Data Collection

Data were collected primarily through face‐to‐face interviews at participants' homes, undertaken by two trained researchers who assessed capacity. Where participants did not have capacity, consultee consent was used. Interviews lasted around two and a half hours, covering a wide battery of measures including key demographics, rates of service use, well‐being, and quality of life. Where the individual with dementia had a participating carer, the carer provided demographic and service‐use data.

### 2.3 Measures

#### 2.3.1 Coping Strategies

The dispositional version of the Brief COPE is a 28‐item measure assessing individuals' usage of 14 distinct coping strategies, with higher scores representing greater use [21]; scores can be averaged across these strategies to measure broader coping styles. The Brief COPE has been tested and validated across populations but seen limited use in the context of dementia [22].

#### 2.3.2 Dementia Symptom Severity

Disease severity at diagnosis was used as one indicators of diagnostic timing. The Standardized Mini‐Mental State Examination (SMMSE) primarily assesses cognitive symptoms [23], while the Clinical Dementia Rating (CDR) provides a more global measure of symptom progression [24]. Lower scores on the SMMSE and higher scores on the CDR indicate more severe symptoms, suggesting a more delayed diagnosis. Specifically, scores on the SMMSE are often categorised as suggesting normal cognition (30‐26), Mild/Early dementia (25‐21), Moderate dementia (20‐11), or severe dementia (10‐0). On the CDR, a score of 0 indicates no dementia, while 0.5, 1, 2, and 3 indicate questionable, mild, moderate, and severe dementia respectively [24].

#### 2.3.3 Temporal Diagnostic Delay

Carers were asked to recall when they first noticed the onset of symptoms; individuals with dementia with no participating carer answered themselves. The difference in months between this date and the clinically recorded date of diagnosis was calculated, with a longer time period indicating a greater temporal delay in diagnosis.

#### 2.3.4 Demographic Variables

Twenty demographic variables were considered for inclusion in our models as potential predictors (see Table 1 and Supporting Information S1: Table S1). These variables were chosen because they have been previously examined in relation to service use or diagnostic interest in dementia [6, 9, 15, 17].

**TABLE 1:** Descriptive characteristics for individuals with dementia and carers.

Variable	Range	Mean (SD)	*N* (%)	Missing (%)
Person with dementia				
Age	48–105	83.31 (7.7)		0.43
Gender: Female			50.59	0.64*
Ethnicity: White british			86.77	0.53
Has qualifications:			73.05	2.78
SMMSE	0–30	22.3 (5.22)		5.78
CDR	0–3	0.82 (0.52)		3.01
Temporal diagnostic delay (months)	0–332	35.39 (36.91)		27.81
Employed			3.08	2.46
Dementia type: Alzheimer's disease			58.86	0.96
Has comorbidities			41.14	0*
Receiving living or disability allowance			38.5	3.32
Receiving pension credit			17.44	10.48
Urban living area (self‐report)			84.21	2.46
Urban living area (output area score)			85.29	0.43
Index of multiple deprivation	1–10	6.29 (2.75)		0.43
Lives alone			26.41	8.88
Marital status: Married			59.93	0.43
Relationship with primary carer: Spouse			59.54	0.14
Recruited pre‐Covid			27.91	0
Carer				
Age	26–98	71.29 (13)		0
Gender: Female			68.92	0.29
Ethnicity: White british			91.68	0
Has qualifications			87.09	0

*Note:* For gender, 2 participants answered ‘Other’—this was treated as missing data to allow for binary analyses. For comorbidities, missing or uncertain answers for the presence of conditions were treated as ‘No’. For CDR and Relationship with primary carer, data only reported for individuals with participating carers. *N* (%) calculated from total non‐missing data.

## 3 Analyses

We first used the Brief COPE to derive the coping styles included as predictors in the main analysis. To do this, we conducted Principal Component Analysis (PCA) on the 14 Brief COPE subscales separately for people with dementia and for carers. We included all participants with complete data on the Brief COPE, resulting in 652 people with dementia (out of 935) and 573 carers (out of 697) (See Supplementary content for analysis of missing data). The Kaiser‐Meyer‐Olkin measure of sampling adequacy and Bartlett's test of sphericity were used to determine that the dataset was appropriate for PCA. Final standardised loadings were generated using correlation matrix extraction with promax rotation. Parallel scree plots were used to determine the optimal number of principal factors to produce. A minimum threshold of 0.3 was used to determine factor loadings [25]. These results were used to compute coping style scores by summing across the relevant Brief COPE items.

Hierarchical linear regression tested whether coping styles predicted diagnostic timing. Step 1 included core demographics (age, gender, ethnicity, qualifications) and any demographics correlated with the outcome. Coping style scores were then added sequentially: Step 2 for individuals with dementia and Step 3 for carers. SMMSE, CDR scores, and temporal delay in months served as outcomes in three separate models. In these analyses, only dyads with complete data on included variables were included, resulting in 459 dyads in the SMMSE model, 466 dyads in the CDR model, and 399 dyads in the temporal delay model. Weighted least squares (WLS) regression was employed for the temporal delay model due to heteroscedasticity. All analyses were conducted in *RStudio* (R version 4.3.1).

## 4 Results

### 4.1 Demographics

Analyses drew upon data from 1632 participants, consisting of 935 individuals recently diagnosed with dementia and 697 of their primary carers (Table 1).

### 4.2 Understanding Diagnostic Timing

Individuals with dementia produced a mean score of 22.3 on the SMMSE, and 0.82 on the CDR, both indicating mild impairment at the time of diagnosis. The mean time between first noticing symptoms and receiving a diagnosis was 35.4 months, although this varied considerably between participants. While SMMSE and CDR scores were strongly correlated with one another (*r* = −0.60, *p* < 0.001), temporal delay correlated with neither SMMSE (*r* = 0.03, *p* = 0.427) nor CDR (*r* = −0.03, *p* = 0.494).

### 4.3 Identification of Coping Styles

**FIGURE 1:**
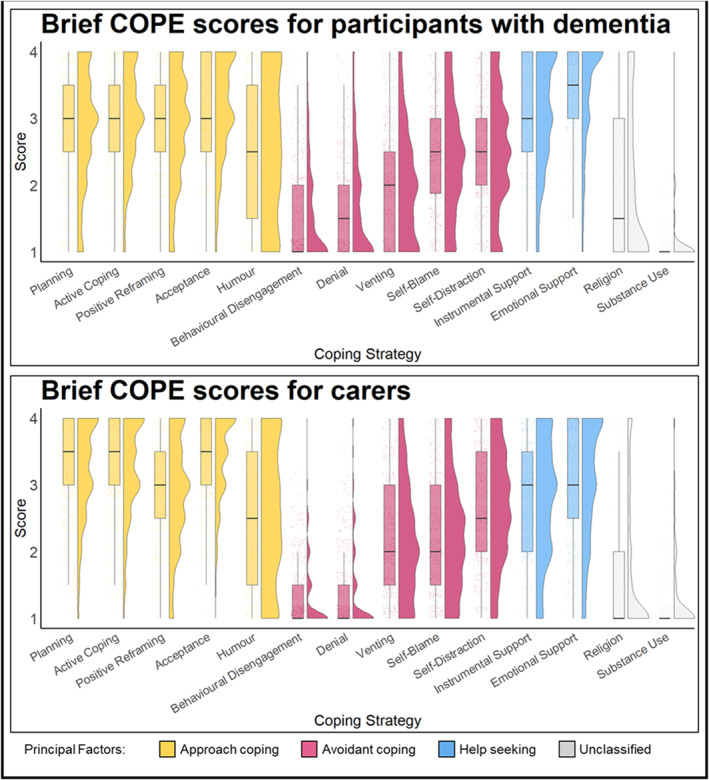
Raincloud plot showing scores on the brief Cope for 652 people with dementia and 573 carers, grouped by coping styles.

The structure of the Brief COPE was examined using principal component analysis. For both samples, a three factorial structure displayed the most adequate psychometric properties, with factors representing traditional groupings of Approach Coping, Avoidant Coping, and Support Seeking (Figure 1). See Supporting Information S1: Table S2, S3 for PCA results.

### 4.4 Predicting Diagnostic Timing—Symptom Severity at Diagnosis

To examine the association between coping and symptom severity at diagnosis, hierarchical linear regression was carried out with SMMSE (Table 2) and CDR (Table 3) serving as outcomes in separate models. Predictors were added over 3 steps, starting with demographics, followed by coping styles for people with dementia and then carers. Step 2 produced the most parsimonious model for both SMMSE scores (*F*(11, 447) = 5.81, *p* < 0.001, adjusted *R* ^2^ = 0.104) and CDR scores (*F*(9, 456) = 7.06, *p* < 0.001, adjusted *R* ^2^ = 0.122). When compared via anova, Step 2 significantly improved upon Step 1 for both SMMSE (*F*(3, 447) = 4.93, *p* = 0.002) and CDR (*F*(3,456) = 3.92, *p* = 0.009) suggesting coping styles of people with dementia were associated with symptom severity at diagnosis beyond demographics alone. The addition of carers' coping styles in Step 3 was insignificant and added no further improvement upon Step 2 for SMMSE (*F*(3,444) = 0.343, *p* = 0.794) or CDR (*F*(3,450) = 0.75, *p* = 0.525).

**TABLE 2:** Hierarchical regression models predicting SMMSE.

Variable	Step 1			Step 2			Step 3		
	*b*	*β*	SE	*b*	*β*	SE	*b*	*β*	SE
Intercept	28.14***		3.36	26.03***		3.72	25.32***		4.21
Age	−0.07*	−0.13	0.03	−0.07*	−0.13	0.03	−0.07*	−0.13	0.03
Gender	−0.59	−0.07	0.44	−0.48	−0.05	0.44	−0.49	−0.06	4.2
Ethnicity	−0.35	−0.02	0.83	−0.30	−0.02	0.82	−0.24	−0.01	0.82
Qualifications	1.26**	0.13	0.44	1.21**	0.13	0.44	1.18**	0.12	0.45
Employment status	−0.85	−0.04	1.09	−0.75	−0.03	1.09	−0.74	−0.03	1.10
Urban area (self‐report)	0.76	0.06	0.56	0.95.	0.08	0.56	0.97.	0.08	0.56
Relationship with carer	−0.43	−0.05	0.48	−0.42	−0.05	0.48	−0.49	−0.06	0.49
Living/disability allowance	0.94*	0.11	0.42	0.66	0.07	0.42	0.69	0.08	0.43
Approach coping (people with dementia)				1.02**	0.14	0.37	1.00**	0.13	0.38
Avoidant coping (people with dementia)				−1.08*	−0.12	0.42	−1.08*	−0.12	0.42
Support seeking (people with dementia)				0.39	0.06	0.31	0.39	0.06	0.32
Approach coping (carer)							0.19	0.02	0.43
Avoidant coping (carer)							−0.20	−0.02	0.47
Support seeking (carer)							0.20	0.04	0.26
*R* ^2^	0.096			0.125			0.127		
Adjusted *R* ^2^	0.080***			0.104***			0.100***		

Abbreviation: SMMSE = Clinical Dementia Rating.

. *p* value < 0.1.

^*^ *p* value < 0.05.

^**^ *p* value < 0.01.

^***^ *p* value < 0.001.

**TABLE 3:** Hierarchical regression models predicting CDR.

Variable	Step 1			Step 2			Step 3		
	*b*	*β*	SE	*b*	*β*	SE	*b*	*β*	SE
Intercept	0.04		0.30	0.04		0.33	0.02		0.37
Age	0.01**	0.15	0.00	0.01**	0.15	0.00	0.01**	0.15	0.00
Gender	0.05	0.05	0.04	0.04	0.04	0.04	0.04	0.05	0.04
Ethnicity	−0.04	−0.02	0.07	−0.05	−0.03	0.07	−0.05	−0.03	0.07
Qualifications	−0.01	−0.01	0.04	0.00	0.00	0.04	0.01	0.01	0.04
Employment status	0.04	0.02	0.10	0.04	0.02	0.10	0.06	0.03	0.10
Dementia type	0.07.	0.09	0.04	0.06	0.07	0.04	0.06	0.07	0.04
Comorbidities	0.02	0.03	0.04	0.02	0.03	0.04	0.02	0.02	0.04
Relationship with carer	0.07	0.08	0.04	0.07	0.08	0.04	0.07.	0.09	0.04
Living/disability allowance	−0.16***	−0.20	0.04	−0.14***	−0.17	0.04	−0.14***	−0.17	0.04
Approach coping (people with dementia)				−0.06.	−0.09	0.03	−0.06.	−0.09	0.03
Avoidant coping (people with dementia)				0.12**	0.14	0.04	0.12**	0.14	0.04
Support seeking (people with dementia)				−0.02	−0.04	0.03	−0.02	−0.04	0.03
Approach coping (carer)							−0.04	−0.05	0.04
Avoidant coping (carer)							0.04	0.05	0.04
Support seeking (carer)							−0.00	−0.01	0.02
*R* ^2^	0.122			0.145			0.149		
Adjusted *R* ^2^	0.105***			0.122***			0.120***		

Abbreviation: CDR = Clinical Dementia Rating.

. *p* value < 0.1.

*p value < 0.05.

^**^ *p* value < 0.01.

^***^ *p* value < 0.001.

As Step 2 produced the most parsimonious models for both symptom severity measures, discussion will be focussed here. Regarding coping styles, more avoidant coping in people with dementia predicted greater symptom severity via SMMSE (*β* = −0.12, *p* = 0.010) and CDR (*β* = 0.14, *p* = 0.002) while more approach coping predicted lower symptom severity via SMMSE (*β* = 0.14, *p* = 0.007).

For demographic variables, greater symptom severity on both measures was predicted by people with dementia being older (SMMSE, *β* = 0.15, *p* = 0.010; CDR *β* = 0.15, *p* = 0.002). Participants having no formal academic qualifications predicted greater symptom severity on the SMMSE (*β* = 0.13, *p* = 0.007) as did receiving living or disability allowance for the CDR (*β* = −0.17, *p* < 0.001).

### 4.5 Predicting Diagnostic Timing—Temporal Delay

As with symptom severity, hierarchical linear regression was used to examine the association between coping and temporal diagnostic delays, defined as the distance in months between reported symptom onset and diagnosis (Table 4). Step 2 again produced the most parsimonious model (*F*(9, 389) = 27.75, *p* < 0.001, adjusted *R* ^2^ = 0.391). Likelihood ratio tests found that the addition of coping scores for participants with dementia in Step 2 significantly improved upon demographics alone in Step 1 (*χ* ^*2*^(3) = 15.21, *p* = 0.002) while adding carers' coping in Step 3 did not improve upon Step 2 (*χ* ^2^(3) = 5.52, *p* = 0.137).

**TABLE 4:** Hierarchical regression models predicting temporal diagnostic delay.

Variable	Step 1			Step 2			Step 3		
	*b*	*β*	SE	*b*	*β*	SE	*b*	*β*	SE
Intercept	59.61***		17.48	90.39***		20.20	80.36***		22.14
Age	−0.43*	−0.09	0.17	−0.39*	−0.08	0.16	−0.34*	−0.07	0.16
Gender	−2.92	−0.04	2.57	−8.61***	−0.12	2.23	−7.25**	−0.10	2.20
Ethnicity	−11.37***	−0.08	2.58	−13.44***	−0.10	1.84	−10.59***	−0.08	2.12
Qualifications	3.78	0.05	2.53	0.45	0.01	2.41	2.95	0.04	2.39
Urban area (self‐report)	12.11.	0.13	6.71	13.81*	0.14	6.79	11.54.	0.12	6.47
IMD	0.99*	0.08	0.46	0.47	0.04	0.43	0.57	0.05	0.43
Approach coping (people with dementia)				−1.50	−0.03	1.50	2.03	0.03	2.12
Avoidant coping (people with dementia)				2.50	0.04	2.56	0.86	0.01	2.67
Support seeking (people with dementia)				−5.30**	−0.10	0.63	−5.84**	−0.11	2.03
Approach coping (carer)							−0.71	−0.01	2.52
Avoidant coping (carer)							−5.49***	−0.07	1.62
Support seeking (carer)							1.99	0.05	1.29
*R* ^2^	0.132			0.391			0.295		
Adjusted *R* ^2^	0.119***			0.377***			0.273***		

Abbreviation: IMD = Index of Multiple Deprivation.

. *p* value < 0.1.

^*^ *p* value < 0.05.

^**^ *p* value < 0.01.

^***^ *p* value < 0.001.

As Step 2 produced the strongest and most parsimonious model, we will primarily focus here. For coping style, greater temporal delay was predicted by individuals with dementia using less support seeking coping (*β* = −0.10, *p* = 0.001). However, carers' avoidant coping also exhibited a significant effect in Step 3, despite the non‐significant change in model fit, with greater avoidant coping predicting less temporal delay (*β* = −0.07, *p* < 0.001). Unstandardised results reveal that a one‐point change in either of these coping styles was associated with a temporal change of over 5 months.

Longer temporal delay was also predicted by individuals with dementia being younger (*β* = −0.08, *p* = 0.015), male (*β* = −0.12, *p* < 0.001), White British (*β* = −0.10, *p* < 0.001), and living in a rural area (*β* = 0.14, *p* = 0.043).

## 5 Discussion

We provide novel evidence that the coping styles of people with dementia can predict diagnostic timing, beyond demographic variables already known to be associated. This evidence draws on a large, diverse cohort of recently diagnosed people with dementia and their carers, using a measure of coping styles generated from a psychometrically robust measure completed shortly after diagnosis, and three measures of diagnostic timing. These findings have valuable implications for current global policy directives aimed at supporting people to access an early diagnosis.

### 5.1 Predicting Diagnostic Timing

In people with dementia, greater use of avoidant coping styles predicted increased symptom severity at diagnosis for both measures of severity, suggesting a later diagnosis; greater use of approach coping predicted decreased symptom severity, although only on the SMMSE. These results expand on previous qualitative investigations that have indicated the delaying impacts of maladaptive strategies such as disengagement and denial [12, 13]. Coping style also predicted our temporal measure of diagnostic timing. Greater use of support seeking predicted shorter delays between symptom recognition and diagnosis, aligning with previous research highlighting the role of support networks in facilitating earlier diagnosis [7].

Less intuitively, greater use of avoidant coping in carers predicted shorter temporal diagnostic delays (i.e., less time between symptom recognition and clinical diagnosis). One possible explanation is that avoidant coping may sometimes amplify the perceived need for a diagnosis. Dysfunctional coping strategies have been linked to greater distress and carer burden [26] and may also prevent carers from accessing other forms of support that could delay the urgency of seeking a diagnosis [18]; whereas if carers are managing well and perceive no need for a diagnosis, it may take longer for one to be sought [14].

Alternatively, carers' coping styles may influence how and when they recognise symptoms. Carers who rely on avoidant strategies, such as denial, may overlook or minimise early signs of dementia until symptoms become too pronounced to ignore [27], therefore reporting a later date of symptom onset. The presence of more severe symptoms at an initial consultation could additionally result in accelerated movement through the healthcare system, further reducing the apparent time to diagnosis. Future research should examine how different measures of diagnostic timing align with one another and take care when relying on individual measures as outcomes.

Our results have important implications for policies targeting early dementia diagnosis. Research identifying factors that may delay engagement with diagnostic and support services is vital to inform policy recommendations and allow awareness campaigns and policies promoting earlier diagnosis to be tailored towards individuals who will benefit most. However, current policy directives are largely focussed on addressing structural changes and external barriers, such as the implementation of new services, and neglect the importance of modifiable psychological factors, particularly for people with dementia. If individuals with higher rates of avoidant coping are at greater risk of experiencing diagnostic delays, more effort and resources must be afforded to future initiatives that seek to address psychological factors influencing help‐seeking behaviour as well as these recognised external barriers.

Our identification of individual coping styles as a crucial determinant of help‐seeking behaviour also has important implications for primary care. Our findings suggest it may be beneficial for clinicians to consider or formally assess coping styles when patients present with cognitive symptoms, to help them tailor the level of support provided throughout the diagnostic process. Individuals with avoidant coping tendencies may require more proactive support to encourage engagement with diagnostic services. Coping may also influence other aspects of help‐seeking post‐diagnosis, in which case support services could be tailored to individuals' coping styles throughout their illness. Future research should further explore how coping impacts help seeking and evaluate targeted interventions in real‐world settings, including a focus on how such initiatives may be best promoted towards individuals with more avoidant coping styles.

Coping styles also have the crucial benefit of being modifiable and already serve as targets within a range of illness management interventions, including for dementia [28]. However, most of these interventions are aimed exclusively towards carers. Indeed, past research emphasised the role of carers in shaping decisions around healthcare, potentially overlooking the agency and influence of individuals with dementia themselves [7]. If diagnostic timing is influenced by the coping styles of individuals with dementia, as our findings suggest, then interventions should also be aimed at promoting adaptive coping in older adults with or at risk of dementia. Such initiatives could not only benefit wellbeing but also contribute to earlier detection and improved disease management if dementia does develop.

### 5.2 Strengths and Limitations

Our study has several strengths. We have drawn on data from a large and diverse sample of carers and individuals with dementia close to the point of diagnosis. Furthermore, this comprehensive data enabled us to control for a wide array of demographic variables to support our conclusion that individual coping styles are associated with diagnostic timing. Additionally, as there is currently no single standard method for defining or measuring diagnostic timing [29], our study benefited from drawing on multiple outcome measures. Finally, coping styles were based on PCA of an established measure of coping, an approach that is rarely undertaken in similar research and which allows us to be confident that these are representative of actual coping styles within our sample.

Study limitations include our use of a dispositional measure of coping, taken post‐diagnosis, to predict diagnostic timing. However, the dispositional Brief COPE has demonstrated high test‐retest reliability in dementia carers [22], with longitudinal interviews suggesting similar stability in individuals with dementia [30], suggesting that scores are likely to accurately reflect pre‐diagnostic coping, especially given the recruitment of participants immediately following diagnosis. Another limitation is that our measure of temporal diagnostic delay relies on carer reports. Although this is mitigated by our use of multiple outcome measures, larger gaps may represent earlier recognition of symptoms as well as later diagnoses. Finally, several of our produced principal components had sub‐optimal internal reliability and we observed moderate rates of missing data.

## 6 Conclusions

Our study provides the first quantitative evidence, using statistically robust measures, that the coping styles of individuals with dementia and their carers predict diagnostic timing over and above sociodemographic characteristics. These findings have important implications for future policy directives and interventions aimed at supporting early diagnosis of dementia. Our research also highlights the role that individuals with dementia play in shaping key decisions about their own healthcare, demonstrating the need for research that incorporates their perspectives rather than focusing solely on carers or the general public.

## Funding

This work was supported by the Economic and Social Research Council (ESRC) and the National Institute for Health Research (NIHR), grant number ES/S010351/1. The funders had no role in study design, data collection, data analysis, data interpretation, or writing of the report.

## Ethics Statement

Ethical approvals were granted by the Health Research Authority Brighton and Sussex Research Ethics Committee (REC 19/LO/0528. IRAS 261263) according to the guidelines of the Declaration of Helsinki.

## Conflicts of Interest

The authors declare no conflicts of interest.

## Supporting information


Supporting Information S1

## Data Availability

Direct access will be granted to authorised representatives from the Sponsor and host institution for monitoring and/or audit of the study to ensure compliance with regulations. All research data will be archived and securely stored for 10 years after the end point of the study. Following the end of the study, anonymised data will also be uploaded to the UK Data Archive online repository. Access to data will be limited to authorised researchers who will agree to the End User Licence (http://dataarchive.ac.uk/conditions).

## References

[1] World Health Organization , “Global Action Plan on the Public Health Response to Dementia 2017–2025,” in Global Action Plan on the Public Health Response to Dementia 2017–2025: [Internet]. (2017): [cited 2025 Oct 20], https://www.who.int/publications/i/item/global‐action‐plan‐on‐the‐public‐health‐response‐to‐dementia‐2017‐2025.

[2] B. Dubois , A. Padovani , P. Scheltens , A. Rossi , and G. Dell’Agnello , “Timely Diagnosis for Alzheimer’s Disease: A Literature Review on Benefits and Challenges,” Journal of Alzheimer’s Disease 49, no. 3 (January 2016): 617–631: [cited 2025 Oct 20], 10.3233/jad-150692. PMC4927869 26484931

[3] J. Rasmussen and H. Langerman , “Alzheimer’s Disease—Why We Need Early Diagnosis,” Degenerative Neurological and Neuromuscular Disease 9 (December 2019): 123–130: [cited 2025 Oct 20], 10.2147/dnnd.s228939. 31920420 PMC6935598

[4] R. Watson , J. Bryant , R. Sanson‐Fisher , E. Mansfield , and T. J. Evans , “What Is a ‘Timely’ Diagnosis? Exploring the Preferences of Australian Health Service Consumers Regarding When a Diagnosis of Dementia Should Be Disclosed,” BMC Health Services Research 18, no. 1 (December 2018): 1–9: [cited 2025 Oct 20], 10.1186/s12913-018-3409-y. 30081889 PMC6080387

[5] S. Gauthier , R. N. Pedro , A. M. José , and W. Claire , “World Alzheimer Report 2021: Journey Through the Diagnosis of Dementia,” Alzheimer's Disease Internationa 30 (2022): [cited 2025 Oct 20], https://www.alzint.org/resource/world‐alzheimer‐report‐2021/.

[6] O. Kusoro , M. Roche , R. Del‐Pino‐Casado , P. Leung , and V. Orgeta , “Time to Diagnosis in Dementia: A Systematic Review With Meta‐Analysis,” International Journal of Geriatric Psychiatry 40, no. 7 (July 2025): e70129, 10.1002/gps.70129. 40716451 PMC12300619

[7] M. Parker , S. Barlow , J. Hoe , and L. Aitken , “Persistent Barriers and Facilitators to Seeking Help for a Dementia Diagnosis: A Systematic Review of 30 Years of the Perspectives of Carers and People With Dementia,” International Psychogeriatrics 32, no. 5 (May 2020): 611–634: [cited 2025 Oct 20], 10.1017/S1041610219002229. 32024558

[8] L. Perry‐Young , G. Owen , S. Kelly , and C. Owens , “How People Come to Recognise a Problem and Seek Medical Help for a Person Showing Early Signs of Dementia: A Systematic Review and Meta‐Ethnography,” Dementia 17, no. 1 (January 2018): 34–60: [cited 2025 Oct 20], 10.1177/1471301215626889. 26764265 PMC5758935

[9] P. Werner , D. Goldstein , D. S. Karpas , L. Chan , and C. Lai , “Help‐Seeking for Dementia: A Systematic Review of the Literature,” Alzheimer Disease and Associated Disorders 28, no. 4 (December 2014): 299–310: [cited 2025 Oct 20], 10.1097/wad.0000000000000065. 25321607

[10] L. Clare , “We’ll Fight it as Long as We Can: Coping With the Onset of Alzheimer’s Disease,” Aging & Mental Health 6, no. 2 (May 2002): 139–148: [cited 2025 Oct 20], 10.1080/13607860220126826. 12028882

[11] C. M. Coppens , S. F. de Boer , and J. M. Koolhaas , “Coping Styles and Behavioural Flexibility: Towards Underlying Mechanisms,” in Philosophical Transactions of the Royal Society of London. Series B, Biological Sciences 365, no. 1560 (2010): 4021–4028: [cited 2025 Oct 20], 10.1098/rstb.2010.0217. 21078654 PMC2992750

[12] S. Devoy and E. E. A. Simpson , “Help‐Seeking Intentions for Early Dementia Diagnosis in a Sample of Irish Adults,” Aging & Mental Health 21, no. 8 (August 2017): 870–878: [cited 2023 Feb 12], 10.1080/13607863.2016.1179262. 27149181

[13] V. A. Grunberg , S. Bannon , P. Popok , M. Reichman , B. C. Dickerson , and A. M. Vranceanu , “A Race Against Time: Couples’ Lived Diagnostic Journeys to Young‐Onset Dementia,” Aging & Mental Health 26, no. 11 (October 2022): 2223–2232: [cited 2025 Oct 20], 10.1080/13607863.2021.1966748. 34435534 PMC9377159

[14] J. Henley , A. Hillman , I. R. Jones , et al., “‘We’re Happy as We Are’: The Experience of Living With Possible Undiagnosed Dementia,” Ageing and Society 43, no. 9 (September 2023): 2041–2066: [cited 2025 Oct 20], 10.1017/S0144686X21001495.

[15] K. D. Christensen , J. S. Roberts , B. J. Zikmund‐Fisher , et al., “Associations Between self‐referral and Health Behavior Responses to Genetic Risk Information,” Genome Medicine 7, no. 1 (December 2015): 1–11: [cited 2025 Oct 20], 10.1186/s13073-014-0124-0. 25642295 PMC4311425

[16] M. Maxfield , R. Cui , J. R. Roberts , and A. Fiske , “Interest in Dementia Testing: Family History, Dementia‐Related Anxiety, and Coping,” GeroPsych 34, no. 1 (2021): 5–11, 10.1024/1662-9647/a000230.

[17] S. Y. Tay , J. Davison , N. C. Jin , and P. L. K. Yap , “Education and Executive Function Mediate Engagement in Advance Care Planning in Early Cognitive Impairment,” Journal of the American Medical Directors Association 16, no. 11 (November 2015): 957–962: [cited 2025 Oct 20], 10.1016/j.jamda.2015.05.014. 26130078

[18] M. Roelands , P. Van Oost , and A. Depoorter , “Service Use in Family Caregivers of Persons With Dementia in Belgium: Psychological and Social Factors,” Health and Social Care in the Community 16, no. 1 (2008): 42–53: [cited 2023 July 15], 10.1111/j.1365-2524.2007.00730.x. 18181814

[19] C. E. Gill , G. A. Hinrichsen , and R. DiGiuseppe , “Factors Associated With Formal Service Use by Family Members of Patients With Dementia,” Journal of Applied Gerontology 17, no. 1 (March 1998): 38–52: [cited 2025 Oct 20], 10.1177/073346489801700103.

[20] N. Farina , B. Hicks , K. Baxter , et al., “DETERMinants of Quality of Life, Care and Costs, and Consequences of INequalities in People With Dementia and Their Carers [DETERMIND]: A Protocol Paper,” International Journal of Geriatric Psychiatry 35, no. 3 (2020): 290–301: [cited 2025 Oct 20], 10.1002/gps.5246. 31876069

[21] C. S. Carver , “You Want to Measure Coping But Your Protocol’ Too Long: Consider the Brief Cope,” International Journal of Behavioral Medicine 4, no. 1 (March 1997): 92–100: [cited 2025 Oct 20], 10.1207/s15327558ijbm0401_6. 16250744

[22] C. Cooper , C. Katona , and G. Livingston , “Validity and Reliability of the Brief COPE in Carers of People With Dementia: The LASER‐AD Study,” Journal of Nervous and Mental Disease 196, no. 11 (November 2008): 838–843: [cited 2025 Oct 20], 10.1097/NMD.0b013e31818b504c. 19008735

[23] D. W. Molloy and T. I. M. Standish , “A Guide to the Standardized Mini‐Mental State Examination,” supplement, International Psychogeriatrics 9, no. S1 (December 1997): 87–94: [cited 2025 Oct 20], 10.1017/S1041610297004754. 9447431

[24] J. C. Morris , “Clinical Dementia Rating: A Reliable and Valid Diagnostic and Staging Measure for Dementia of the Alzheimer Type,” International Psychogeriatrics 9 (December 1997): 173–176: [cited 2025 Oct 20], 10.1017/S1041610297004870. 9447441

[25] K. Baumstarck , M. Alessandrini , Z. Hamidou , P. Auquier , T. Leroy , and L. Boyer , “Assessment of Coping: A New French Four‐Factor Structure of the Brief COPE Inventory,” Health and Quality of Life Outcomes 15, no. 1 (January 2017): 1–9: [cited 2025 Oct 20], 10.1186/s12955-016-0581-9. 28077139 PMC5225566

[26] C. Zucchella , M. Bartolo , C. Pasotti , L. Chiapella , and E. Sinforiani , “Caregiver Burden and Coping in Early‐Stage Alzheimer Disease,” Alzheimer Disease and Associated Disorders 26, no. 1 (March 2012): 55–60: [cited 2025 Oct 20], 10.1097/WAD.0b013e31821aa6de. 21537145

[27] K. K. Leung , J. Finlay , J. L. Silvius , et al., “Pathways to Diagnosis: Exploring the Experiences of Problem Recognition and Obtaining a Dementia Diagnosis Among Anglo‐Canadians,” Health and Social Care in the Community 19, no. 4 (July 2011): 372–381, 10.1111/j.1365-2524.2010.00982.x. 21223398

[28] K. J. Gilhooly , M. L. M. Gilhooly , M. P. Sullivan , et al., “A Meta‐Review of Stress, Coping and Interventions in Dementia and Dementia Caregiving,” BMC Geriatrics 16, no. 1 (December 2016): 1–8: [cited 2025 Oct 20], 10.1186/s12877-016-0280-8. 27193287 PMC4872341

[29] Y. Chen , M. C. Power , F. Grodstein , et al., “Correlates of Missed or Late Versus Timely Diagnosis of Dementia in Healthcare Settings,” Alzheimer's & Dementia 20, no. 8 (2024): 5551–5560: [cited 2025 Oct 20], 10.1002/alz.14067. PMC11350028 38934297

[30] K. Thorsen , M. C. N. Dourado , and A. Johannessen , “Awareness of Dementia and Coping to Preserve Quality of Life: A Five‐Year Longitudinal Narrative Study,” International Journal of Qualitative Studies on Health and Well‐Being 15, no. 1 (January 2020): 1798711: [cited 2023 June 7], 10.1080/17482631.2020.1798711. 32780653 PMC7482873

