# Supplementary material for: Coping Styles Predict Timing of Dementia Diagnosis: Evidence From DETERMIND
Source: Int J Geriatr Psychiatry. 2026 Jul 31;41(8):e70246. doi: 10.1002/gps.70246 (PMC13426043; doi:10.1002/gps.70246)
Supplement: Supplementary file 1 — Supporting Information S1 [file GPS-41-e70246-s001.docx]

# Coping styles predict timing of dementia diagnosis: evidence from DETERMIND.

## Supplementary Data

### Demographic Variables

| **Table S1** | | | |  | |
| --- | --- | --- | --- | --- | --- |
| **Demographic Variables Considered for Initial Regression Model** | | | |  | |
| Variable |  | Levels of binary variables | | | |
|  |  | Level 1 |  | Level 2 |  |
| Age (years) |  |  |  |  |  |
| Gender |  | Male |  | Female |  |
| Ethnicity |  | White British |  | Other |  |
| Qualifications |  | No qualifications |  | Any qualifications |  |
| Employment status |  | Employed |  | Unemployed |  |
| Marital status |  | Married |  | Not married |  |
| Dementia Type |  | Alzheimer’s Disease |  | Other dementia |  |
| Comorbidities |  | None reported |  | Has comorbidities |  |
| Relationship with primary carer |  | Spouse |  | Other relationship |  |
| Receiving living or disability allowance |  | Receiving either |  | Receiving neither |  |
| Receiving pension credit |  | Receiving |  | Not receiving |  |
| Urban living area (self-reported) |  | Urban |  | Rural |  |
| Urban living area (output area score) |  | Urban |  | Rural |  |
| Covid Cohort |  | Recruited prior |  | Recruited after |  |
| Living alone |  | Lives alone |  | Lives with others |  |
| Index of Mass Deprivation (1-10) |  |  |  |  |  |
| Age, gender, ethnicity, and qualifications were included for participants with dementia and carers | | | | | |

### Analysis of Missing Data

A substantial number of participants were initially excluded due to missing data from their Brief COPE responses. Following this, remaining rates of missing data were moderate, and more substantial for certain variables such as pension credit status or the date symptoms were first noticed, necessary for calculating temporal diagnostic delay. To examine the potential impact of missing data, individuals with missing data for any variables used in regression analyses were compared to those with complete data. Independent t-tests were used to determine that participants with missing data had more severe symptoms on average when measured via SMMSE (*t*(455) = -5.57, *p* <.001) and CDR (*t*(468) = 5.07, *p* <.001). Those with missing data had an average SMMSE score of 20.68, compared to 23.13 for those with complete data; for the CDR this was 0.94 and 0.72 respectively. Chi-squared tests revealed that the presence of missing data also varied across ethnicity (*X^2^* (1) = 5.55, *p* = .019), with 7% of participants with complete data being non-white, compared to 12.5% for those with missing data.

### Principal Component Analysis Results

Principal component analysis was conducted on the Brief COPE for both people with dementia and for carers. The 3 factor solution produced by our PCA of the Brief COPE closely resembled traditional groupings of approach, avoidant, and support seeking coping. A 3-factor reduction was found to be among the most common in other populations (Solberg et al., 2022), although several other studies grouped strategies slightly differently to ours, in line with emotion-focused, problem-focused, and dysfunctional coping styles. In a study of dementia carers by Roopalekha Jathanna et al., (2010), PCA of the Brief COPE instead produced a six-factor solution, though limited details are provided.

Produced coping styles explained 48.98% of the variance for people with dementia and 48% of the variance for carers with approach coping explaining the most variance, while support seeking explained the least. Neither substance use nor religion loaded onto produced factors and were removed during PCA, resulting in improved model fit. Neither strategy was found to correlate with any of our outcome measures, with rates of substance use likely too low for meaningful analysis.

People with dementia and carers reported similar use of coping strategies (Figure 1). Approach and support seeking strategies (such as planning, active coping, and seeking emotional support) were more commonly used than avoidant strategies (such as behavioural disengagement, denial, and substance use). However, except for substance use, use of each strategy varied moderately across participants

For people with dementia, Cronbach α scores were: 0.70 for approach coping, 0.55 for avoidant coping and 0.70 for support seeking. For carers, Cronbach α scores were: 0.63 for approach coping, 0.53 for avoidant coping and 0.74 for support seeking. See Table S2 and Table S3 for principal factor loadings, explained variance, and eigenvalues. It should also be noted that for both generated avoidant subscales, Cronbach α scores were below 0.6, suggesting questionable internal consistency. This can also be seen in the increased variation of scores reported across specific coping strategies within the avoidant subscale. Many participants reported using self-distraction (“turning to other activities…”) while very few reported using the semantically similar though more severe strategy of behavioural disengagement (“giving up trying to deal with it”). Indeed, more avoidant subscales often produce the lowest internal consistency within the Brief-COPE, both within the original subscales (Bautista et al., 2013) and in generated superordinate scales (Baumstarck et al., 2017).

| **Table S2** | | | | |
| --- | --- | --- | --- | --- |
| **Principal Factor Loadings, Variance Explained, and Eigenvalues for Promax**  **Rotated Solution for 14 Brief COPE Items in 652 Individuals with Dementia** | | | | |
|  | Factor Loading | | |  |
|  | 1 | 2 | 3 | Communality |
| Planning | **0.84** |  |  | 61% |
| Active Coping | **0.77** |  |  | 53% |
| Positive Reframing | **0.69** |  |  | 53% |
| Acceptance | **0.60** |  |  | 37% |
| Humour | 0.48 |  |  | 29% |
| Behavioural Disengagement |  | **0.74** |  | 51% |
| Denial |  | **0.71** |  | 47% |
| Venting |  | **0.60** |  | 40% |
| Self-Blame |  | 0.51 |  | 35% |
| Self-Distraction |  | 0.35 |  | 30% |
| Instrumental Support |  |  | **0.91** | 76% |
| Emotional Support |  |  | **0.89** | 76% |
|  |  |  |  |  |
| Variance Explained | 20.63% | 14.77% | 13.59% |  |
| Eigen Values | 2.48 | 1.77 | 1.63 |  |
| Factors ≥ 0.6 in bold, factors ≤ 0.3 removed | | | | |

| **Table S3** | | | | |
| --- | --- | --- | --- | --- |
| **Principal Factor Loadings, Variance Explained, and Eigenvalues for Promax**  **Rotated Solution for 14 Brief COPE Items in 573 Dementia Carers** | | | | |
|  | Factor Loading | | |  |
|  | 1 | 2 | 3 | Communality |
| Active Coping | **0.72** |  |  | 0.54 |
| Planning | **0.70** |  |  | 0.50 |
| Acceptance | **0.69** |  |  | 0.44 |
| Positive Reframing | **0.64** |  |  | 0.48 |
| Humour | 0.45 |  |  | 0.30 |
| Behavioural Disengagement |  | **0.68** |  | 0.50 |
| Self-Blame |  | **0.62** |  | 0.38 |
| Denial |  | 0.58 |  | 0.35 |
| Venting |  | 0.57 |  | 0.35 |
| Self-Distraction |  | 0.49 |  | 0.38 |
| Instrumental Support |  |  | **0.88** | 0.78 |
| Emotional Support |  |  | **0.87** | 0.77 |
|  |  |  |  |  |
| Variance Explained | 18.91% | 15.47% | 13.62% |  |
| Eigen Values | 2.27 | 1.86 | 1.63 |  |
| Factors ≥ 0.6 in bold, factors ≤ 0.3 removed | | | | |

## Supplemental references:

Solberg, M. A., Gridley, M. K., & Peters, R. M. (2022). The Factor Structure of the Brief Cope: A Systematic Review. *Western Journal of Nursing Research*, *44*(6), 612–627. https://doi.org/10.1177/01939459211012044

Roopalekha Jathanna, P., Latha, K., & Bhandary, P. (2010). Burden and Coping in Informal Caregivers of Persons with Dementia: A Cross Sectional Study. *Online Journal of Health and Allied Sciences*, *9*(4). https://www.ojhas.org/issue36/2010-4-7.htm

Bautista, R. E. D., Rundle-Gonzalez, V., Awad, R. G., & Erwin, P. A. (2013). Determining the coping strategies of individuals with epilepsy. *Epilepsy & Behavior*, *27*(2), 286-291. https://doi-org.sussex.idm.oclc.org/10.1016/j.yebeh.2013.01.029

Baumstarck, K., Alessandrini, M., Hamidou, Z., Auquier, P., Leroy, T., & Boyer, L. (2017). Assessment of coping: A new french four-factor structure of the brief COPE inventory. *Health and Quality of Life Outcomes*, *15*(1), Article 1. https://doi.org/10.1186/s12955-016-0581-9
